# Design Validation of a Relational Agent by COVID-19 Patients: Mixed Methods Study

**DOI:** 10.2196/42740

**Published:** 2023-06-08

**Authors:** Ashraful Islam, Beenish Moalla Chaudhry

**Affiliations:** 1 School of Computing and Informatics University of Louisiana at Lafayette Lafayette, LA United States; 2 Center for Computational and Data Sciences Independent University, Bangladesh Dhaka Bangladesh; 3 Department of Computer Science and Engineering Independent University, Bangladesh Dhaka Bangladesh

**Keywords:** COVID-19, relational agent, mHealth, design validation, health care, chatbot, digital health intervention, health care professional, heuristic, health promotion, mental well-being, design validation survey, self-isolation

## Abstract

**Background:**

Relational agents (RAs) have shown effectiveness in various health interventions with and without doctors and hospital facilities. In situations such as a pandemic like the COVID-19 pandemic when health care professionals (HCPs) and facilities are unable to cope with increased demands, RAs may play a major role in ameliorating the situation. However, they have not been well explored in this domain.

**Objective:**

This study aimed to design a prototypical RA in collaboration with COVID-19 patients and HCPs and test it with the potential users, for its ability to deliver services during a pandemic.

**Methods:**

The RA was designed and developed in collaboration with people with COVID-19 (n=21) and 2 groups of HCPs (n=19 and n=16, respectively) to aid COVID-19 patients at various stages by performing 4 main tasks: testing guidance, support during self-isolation, handling emergency situations, and promoting postrecovery mental well-being. A design validation survey was conducted with 98 individuals to evaluate the usability of the prototype using the System Usability Scale (SUS), and the participants provided feedback on the design. In addition, the RA’s usefulness and acceptability were rated by the participants using Likert scales.

**Results:**

In the design validation survey, the prototypical RA received an average SUS score of 58.82. Moreover, 90% (88/98) of participants perceived it to be helpful, and 69% (68/98) of participants accepted it as a viable alternative to HCPs. The prototypical RA received favorable feedback from the participants, and they were inclined to accept it as an alternative to HCPs in non-life-threatening scenarios despite the usability rating falling below the acceptable threshold.

**Conclusions:**

Based on participants’ feedback, we recommend further development of the RA with improved automation and emotional support, ability to provide information, tracking, and specific recommendations.

## Introduction

### Background

A relational agent (RA) is an artificial intelligence (AI)–based computational artifact that is responsible for maintaining a virtual socioemotional relationship with the user for a long time [1]. According to Bickmore and Cassell [2], with an RA, “It’s just like you talk to a friend.” An RA is a special type of conversational agent (CA), which is a conversation system that can process natural language and respond using human language. CAs are typically implemented as chatbots on the internet or as personal assistants on smartphones or wearable devices. The CA’s interaction with the user does not have to be limited to text. Embodiment of empathy and tangible relational affects transform a CA into an embodied conversational agent (ECA), which is a computer-generated virtual person with animated gestures to facilitate face-to- face interactions between a person and a computer. RAs can also be embodied (embodied RAs) allowing them to use both verbal and nonverbal relational affects over an extended time to form long-term, deep, and meaningful connections with the users. The major difference between RAs and CAs is that, unlike RAs, CAs do not have the capacity to maintain long-term relationships with their users.

Both CAs and RAs have been used for different health care services such as screening, counseling, and caregiving [3-5] in diverse health care settings. Since similar health care services are essential for COVID-19 patients [6-8], CAs and RAs can also be used to support SARS-CoV-2–infected individuals. Moreover, they can remotely deliver essential health services [9], minimizing face-to-face interactions and preventing the transmission of infection. However, in the context of COVID-19, an RA can be more effective than a CA due to its potential to not only offer support and guidance but also maintain a sustained relationship with the user throughout the self-isolation period. A range of CAs was developed during the COVID-19 pandemic, but, to the best of our knowledge, there is no RA that can deliver health care services to COVID-19 patients in non-life-threatening situations.

### Objective

The goal of this research was to address this gap by designing an RA-based intervention that can help patients as they go through different stages of COVID-19. In this paper, we present the early usability, usefulness, and acceptance evaluation of a prototypical RA that was designed for COVID-19 patients using a user-centered design (UCD) approach.

### Related Work

The majority of virtual agent–based interventions that have been proposed during the COVID-19 pandemic are modeled as CAs, used chatbots, and are capable of performing specific tasks. Some helped patients perform guided symptom checking (eg, [10-12]), while some helped provide mental health interventions [8,13].

Ouerhani et al [14] proposed a cloud-based mobile CA for anxiety-emotion assistance in postquarantine situations during COVID-19 that helped increase consciousness of the real danger of the outbreak. They used natural language understanding (NLU) to analyze and create encouragement among people in infected areas. Also, Welch et al [8] presented an expressive CA that uses automated counseling or motivational interviewing to guide an individual suspected of COVID-19 to reduce stress and inspect thoughts and feelings. Another work by Loveys et al [6] presented a randomized pilot trial of a digital human named “Bella,” a form of virtual assistant (VA) that remotely delivered both stress and loneliness interventions during COVID-19. Completing cognitive behavioral and positive psychology tasks with Bella on a website was part of the intervention. Loneliness, stress, and psychological well-being were all addressed in the activities. Bella was regarded as credible in terms of appearance (human-like facial expression), interpersonal abilities (friendly companionship, nonjudgmental attitude), and information delivery based on the opinions of 30 participants.

Battineni et al [15] proposed an AI-enabled chatbot that can serve patients remotely via awareness and virus updates. It can also provide counseling to help patients recover from psychological damage caused by stress and fear due to the pandemic. Woo et al [16] created “Akira,” an AI-enabled CA, which is close to the work by Battineni et al [15]. It was trained by a deep neural network model, with an accuracy of 90.6% to engage and respond appropriately in 7 forms of pandemic-related conversations, such as mental well-being, cold and flu, medications, and drugs. Akira was tested by 57 people, each of whom came up with a list of 5 questions to ask, and the user experience evaluation revealed that a larger training data set was warranted for better performance.

“Chloe,” a Canadian digital information VA [17], can be portrayed as a benchmark for infodemic management during the COVID-19 pandemic. Chloe would inquire about a user’s symptoms, location, previous travel history, and recent contacts. This evaluation resulted in a tailored suggestion that included connections to local resources such as the COVID-19 recommendations by the user’s province government. Ventoura et al [7] developed an empathy-driven CA named “Theano” that speaks Greek and supports both voice and chat interactions with its users. Theano provides COVID-19 data and facts to users, as well as ideal health practices and the most recent COVID-19–related guidelines. In addition, Theano assists end users in the self-evaluation of their symptoms and guiding them to first-line health care professionals (HCPs). Although Chloe and Theano were responsible for delivering information and guidance, “Jennifer” [18] is another CA that combats misinformation about COVID-19. It answers questions about the COVID-19 pandemic by providing conveniently available and reliable information from reputable sources. It includes a wide range of areas, from case statistics to illness prevention and management best practices.

## Methods

### Proposed System

**Figure 1:**
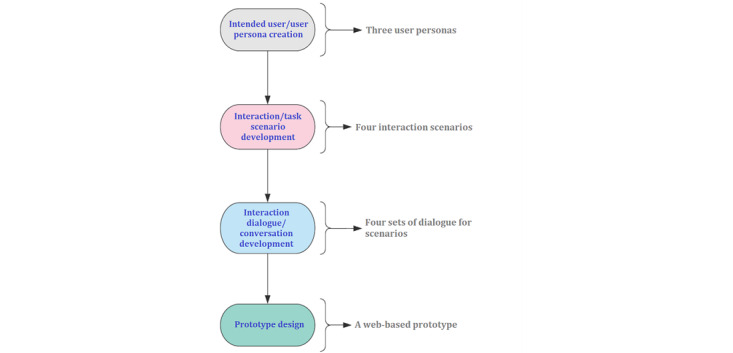
Sequential stages of overall design process from requirement analysis to prototype development.

We used a UCD approach to develop the proposed RA. Figure 1 illustrates the overall design process.

### Intended Users

#### Overview

The intended user groups for the RA were identified through a literature review, which consisted of a review on COVID-19 prevention guidelines published by the US Centers for Disease Control and Prevention (CDC) [19] and other related manuscripts published in academic journals. An interview with 19 HCPs who had cared for COVID-19 patients during the pandemic was later conducted to confirm initial findings [20]. This led to the identification of 3 user groups (personas) defined in the following sections. An individual named Oli is used to represent each group. We assume Oli has no underlying illness nor medical conditions, and Oli owns a smartphone.

#### (1) Suspecting Infection

Oli has been experiencing fever, cough, and shortness of breath for many days. Oli is worried that Oli might have caught the SAR-CoV-2 virus but does not want to go for a test unless Oli is sure that it is required. Oli wants to know where to go and what precautions to take in the meantime.

#### (2) Quarantining at Home

Oli has been diagnosed with COVID-19, but the symptoms are mild. Oli has been advised by the doctors to self-isolate at home for at least 14 days and practice health-promoting behaviors such as consuming a well-balanced diet, getting adequate sleep, and taking prescription drugs. The doctor also tells Oli that Oli can benefit from periodic medical check-ins and vital sign monitoring. Oli also believes that Oli can benefit from someone who can provide support during emergency situations.

#### (3) Recovering After Infection

Oli has recently recovered from severe COVID-19, which required admission to the hospital and a week in the intensive care unit (ICU). Although Oli is now back at home and recovered from the infection, the memories of the stay at the ICU and a near-death experience are still fresh in Oli’s mind. Witnessing other patients dying in the hospital, the helplessness of the health care workers, and the overall hospital atmosphere have deeply impacted Oli’s psyche. In addition, Oli is experiencing weakness due to the physical damage caused by the virus. Oli feels that people around Oli do not understand Oli’s feelings, and hence, Oli needs emotional support.

### User Survey and Task Scenarios

#### Survey

To identify the intended goals of the target users, we conducted an online survey with individuals (n=21) who had been through the SARS-CoV-2 infection and recovery stages [20]. The participants were asked to enumerate challenges and difficulties they faced during different stages of the disease as well as the support they required to overcome those challenges. The analysis of the survey responses led to the identification of 4 distinct tasks for which the proposed RA needed to be designed (Table 1).

**Table 1:** Identified task scenarios, categorized by each user persona, that the relational agent (RA) should address as a health service delivery intervention.

User persona	RA’s task scenario
Suspecting infection	Testing guidance
Quarantining at home	Focusing on recovery Handling emergencies
Recovering after infection	Postinfection care

#### (1) Testing Guidance (Scenario 1)

The RA provides testing guidance to Oli when an infection is suspected, by periodically engaging in a dialog to obtain up-to-date symptom status and health metrics.

#### (2) Wellness Support (Scenario 2A)

The RA provides wellness tips and companionship to Oli during self-isolation at home. The RA also monitors Oli’s symptoms to avert and prevent emergencies.

#### (3) Handling Emergencies (Scenario 2B)

The RA monitors Oli’s symptoms to avoid and prevent emergencies. Whenever Oli reports an emergency, such as shortness of breath, the RA takes appropriate steps to detect critical situations and connects Oli with the emergency services of a nearby hospital.

#### (4) Postinfection Care (Scenario 3)

The RA provides companionship and mental health counseling to help Oli recover from the stress of the infection during the recovery phase. The RA attempts to engage Oli in daily activities so that Oli can resume Oli’s pre-infection life.

Since a COVID-19 patient goes through various stages of infection, long-term relationships can help patients successfully navigate this journey. In this work, we present an RA that supports and serves people from the very beginning of their infection (ie, testing guidance) until their postinfection recovery phase (ie, postinfection care). By offering health care advice and required help, the RA functions as a virtual HCP and social companion who attempts to establish a prolonged socioemotional relationship.

### Conversation Design

The dialog script for each task scenario was prepared in consultation with HCPs (n=16) who were recruited from the authors’ online social networks. Sample scripts corresponding to relevant task scenarios were first prepared and then sent to the HCPs for feedback via online surveys. Based on thematic analysis, we identified 3 major characteristics required for the dialog scripts: (1) robust and validated screening algorithm, (2) focus on building trust by validating feelings and setting realistic expectations, and (3) long-term relationship building by frequent check-ins, peer support, and psychological interventions. Moreover, the dialogs were influenced by existing diagnosis and coaching models (eg, COVID-19 diagnosis model by CDC [21], posttraumatic stress disorder [PTSD] reduction methods [22]). The details have been reported in a separate publication [23].

### Prototype

**Figure 2:**
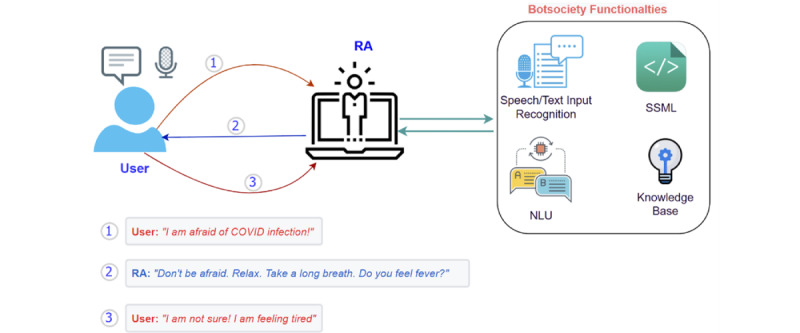
A simplified architecture of the developed prototype in which interactions between the proposed relational agent (RA) and the user take place in human-like natural language. NLU: natural language understanding; SSML: Speech Synthesis Markup Language.

We developed our prototype using a web-based real-time service called BotSociety [24]. The prototypes developed using BotSociety utilize the XML-based Speech Synthesis Markup Language (SSML) to process natural language and produce responses. BotSociety’s NLU module understands user input obtained via a speech or text recognition module, by comparing it with the information stored in its knowledge base. The computing architecture for BotSociety*-*based prototypes is summarized in Figure 2. BotSociety prototypes can be distributed for testing and evaluation purposes via a hyperlink, allowing them to be used on any platform connected to the internet via a web browser. This flexibility makes it possible to test user experience on different systems including smartphones, tablet computers, and desktop or laptop computers.

The RA provides verbal and visual (eg, prompts, graphics, animations) information to the user. The user inputs data using voice or touch and clicks. For improved understanding of the communication between the RA and user, all voice interactions are also displayed as text on the RA’s interface. The user can choose to run the prototype on any of the 3 system modes (ie, smartphone, voice assistant, and tablet computer). We used the scripts that were developed in collaboration with the HCPs (domain experts) from the conversation design phase to create the knowledge base of our prototype for each of the task scenarios available in Table 1.

**Figure 3:**
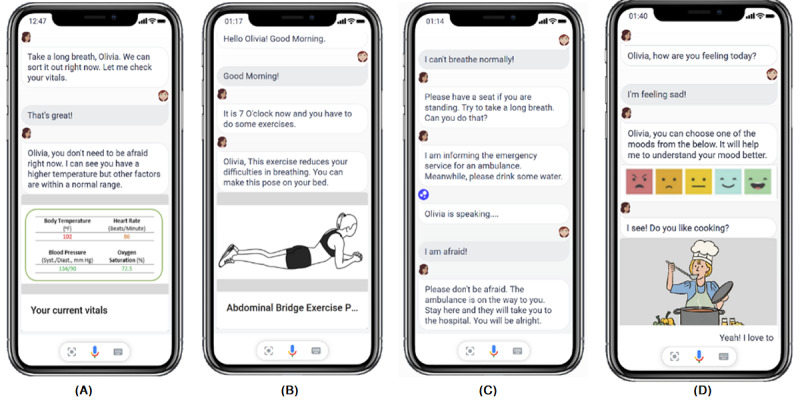
Snippets from the prototype interface is presented in the smartphone view, in which the relational agent (A) checks and shows Oli's physiological vitals and diagnoses for any COVID-19 symptoms (Scenario-1), (B) helps maintain daily healthy practices for fast recovery while Oli is in home isolation (Scenario-2A), (C) takes action during an emergency condition of Oli and tries to make Oli confident by providing affirmative responses (Scenario-2B), and (D) attempts to engage Oli in daily activities to reduce her posttraumatic stress disorder (Scenario-3).

Botsociety provides a flow-based interface for conversation design and a rule-based system that is based on an IF-ELSE dependency mechanism to manage hand-crafted conversations [25] for dialog management. All responses within a category belong to a single topic, and a category is also referred to as a frame. The dependencies allow the RA to shift from one frame to another to ensure the consistency and flow of the conversation. For example, let us assume that the RA wants to coach a user about healthy diet but the user is not interested; the RA will immediately shift the conversation to another frame that is better aligned with user’s current interests. We used the conversation scripts. For example, if a user asks the RA, “Is shortness of breath a symptom of COVID-19?”, the RA will respond “Yes! it is.” and not provide any unnecessary information. The user’s SARS-CoV-2 infection status was checked at the beginning of each interaction. Moreover, an explanation is provided to the user that the RA will continue to record participants’ symptoms and interactions (responses to questions) to continue the refinement of its personalization algorithm. For demonstration purposes in this article, Figure 3 illustrates a few snippets from the developed prototype in smartphone view.

### Prototype Evaluation

#### Goals

Since we sought to validate the proposed RA design, the goal of this evaluation study was to (1) evaluate the usability of the proposed system, (2) determine people’s perceptions about the RA’s usefulness, (3) determine whether people would accept the RA as an alternative to HCPs in non-life-threatening COVID-19 scenarios, (4) elicit people’s preferences regarding the platforms and devices on which they would want to operate the RA, and (5) improve the system design by obtaining feedback from the participants (design recommendations). The study was targeted toward currently infected and recovered individuals so that all the features or scenarios could be evaluated based on participants’ infection experience and postrecovery constraints. The hyperlink to the developed prototype was included in the survey so that the participants could access it through any web browser available on their smartphones or computers. This evaluation study was conducted online, allowing participants to complete the survey at their convenience. There was no restriction on experiencing the prototype, and the participants were free to access it multiple times. The survey was designed in Google Forms, and the order of tasks in this study was controlled. A hyperlink to the survey was created so that the participants could take part in the study by that hyperlink.

#### Participant Recruitment

To ensure diversity of participants in our study, we posted the survey link on various social media platforms (such as Facebook) and mailing lists. The recruitment had no geographical constraints; therefore, individuals from all over the world could take part in this study. The inclusion criteria were (1) being at least 18 years old, (2) previous or current infection with SARS-CoV-2, (3) a basic understanding of the English language since the prototype was developed in English, and (4) having a minimum knowledge of operating a computer to explore the prototype. Participation was voluntary; there was no restriction on who participated, and no identifying information such as name, email address, nationality, or location was collected. The survey took 45 minutes to 60 minutes to complete, and no incentives were offered for completing the survey. All the participants interacted with the RA through all the available modalities (voice, text, click and touch, prompts).

Our recruitment resulted in 98 (53 men) responses from individuals between 18 years and 64 years old, with a mean of age of 34.42 (SD 11.46) years (n=45, 18-30 years; n=25, 31-40 years; n=17, 41-50 years; n=11, 51-64 years). At the time of the survey, 52 participants were infected (n=25, severe symptoms; n=27, mild symptoms), and 46 participants had recovered from the infection (n=17, severe symptoms; n=29, mild symptoms) on the basis of participants’ self-declarations. Everyone owned a smartphone; 64 participants were Android users, and the remaining were iPhone users. Everyone held at least a high school diploma.

#### Study Design

We used a survey-based evaluation to elicit both quantitative and qualitative feedback about the RA from the participants. The survey began with a brief description of the study and informed consent. Participants were then asked to provide necessary demographics (eg, gender, age range, education level, occupation), infection status (currently infected or recovered), and symptom severity. After completing the demographics section, participants completed 4 study tasks, corresponding to each interaction task scenario (Table 1). Each study task involved, first, reading the patient persona description and then interacting with the developed prototype using provided prompts. In other words, participants were asked to pretend that they were using the RA as the presented patient persona to get help. The prompts and link to the prototype were provided within the survey. After completing the task, participants returned to the survey to respond to a series of questions. Specifically, participants indicated the usefulness of the RA for each scenario on a Likert scale and made suggestions (qualitative) for improving the interaction. Each task took 5 minutes to 10 minutes to complete.

After completing all the tasks, participants completed the System Usability Scale (SUS) [26] and indicated their acceptance of the system on a Likert scale. Participants were also asked to specify on which platforms (eg, smartphones, voice assistants, computers) they would prefer to use the RA.

#### Measures and Analysis

Different types of survey responses were collected, such as Likert scale responses, yes or no responses, and qualitative data.

We used the SUS to determine the usability of the presented prototype. The scale was chosen because it is simple to compute and interpret the relative usability and satisfaction with a system. This will also provide a basis for comparison at a later stage of system development.

Microsoft Excel was used to conduct descriptive analysis of the closed questions, which included calculation of means, SDs, percentages, and frequency distributions. The qualitative user feedback was analyzed using the thematic analysis technique [27] using Microsoft Excel. Inductive and deductive coding methods, such as open coding and memoing, were used to code the survey responses after the researchers independently read and reread each response. The researchers then compared their codes and had a discussion to resolve any discrepancies. Related codes were grouped to create minor themes, which were then refined over several iterations and categorized under major themes at the end.

#### Ethics Approval

The institutional review board of the University of Louisiana at Lafayette approved the corresponding user studies (Reference: SP21-82 CACS). All the user studies were conducted online, and the participants responded anonymously. At the beginning of the survey, the study’s objective was stated clearly, and participants could only enter the actual survey after they had indicated a willingness to participate by answering yes to the first question.

## Results

### System Usability

The mean score of each SUS item is presented in Table 2. The overall average SUS score was 58.82 (SD 10.92). The average SUS score (59.88, SD 11.99) of mildly infected participants was slightly higher than that (57.75, SD 9.89) of severely infected participants. However, the difference between the scores of both groups was not statistically significant according to the Student *t* test (*P*=.06).

**Table 2:** Categorized average System Usability Scale (SUS) scores among participants.

SUS item	Mild symptoms, mean	Severe symptoms, mean	Overall sample, mean
I think that I would like to use this system frequently.	4.3	4.05	4.18
I found the system unnecessarily complex.	3.35	3.05	3.2
I thought the system was easy to use.	3.95	3.85	3.9
I think that I would need the support of a technical person to be able to use this system.	3.05	3.5	3.28
I found the various functions in this system were well-integrated.	3.95	3.65	3.8
I thought there was too much inconsistency in this system.	3.3	3.2	3.25
I would imagine that most people would learn to use this system very quickly.	4.0	4.0	4.0
I found the system very cumbersome to use.	3.25	3.55	3.4
I felt very confident using the system.	4.15	4.05	4.1
I needed to learn a lot of things before I could get going with this system.	3.45	3.2	3.33
SUS score	59.88 (11.99)^a^	57.75 (9.89)^a^	58.82 (10.90)^a^

^a^Mean (SD).

### Model’s Usefulness

**Figure 4:**
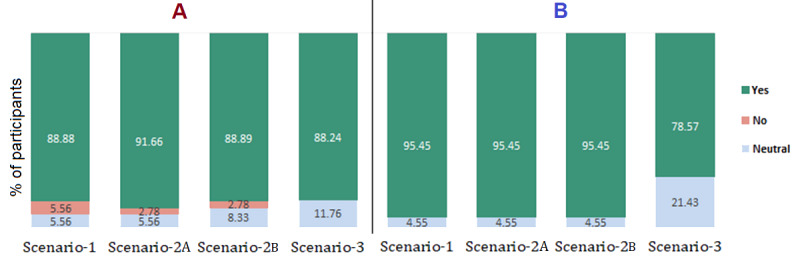
Chart illustrating the details of participants' votes (on a 3-point Likert scale) on model usefulness categorized by infection severity: (A) mild symptoms and (B) severe symptoms. Scenario-1: testing guidance; Scenario-2A: wellness support; Scenario-2B: handling emergencies; Scenario-3: postinfection care.

The usefulness of the RA was determined based on participants’ responses to the following question for each scenario and the overall system: “Indicate your degree of agreement with the following: The services provided by the RA are useful for the target persona.” Participants’ ratings of the usefulness of each RA task are presented in Figure 4. For each task (scenario), most of the participants agreed that the system is very useful or useful. Scenario 3 received the smallest number of usefulness votes by both mildly and severely infected participants. There was no severely infected participant who disagreed with the usefulness of any one of the RA tasks. For the overall system, 90% (88/98; SD 7%; severe symptoms: 40/42, 96%; mild symptoms: 48/56, 86%) of the participants thought that the RA is useful. However, among the participants, 9% (9/98; SD 3%; severe symptoms: 2/42, 5%; mild symptoms: 4/56, 8%) were unsure about the model’s usefulness, and 2% (2/98; SD 2%; only mild symptoms: 2/56, 3%) did not think the model was useful.

### Acceptable Alternative

The acceptance of the proposed RA was measured by participants’ responses to the following question: “Would you accept the proposed RA as an alternative to caregivers/HCPs in non-life-threatening situations?”

Overall, 69% (68/98; SD 6%) of participants (mild symptoms: 40/56, 72%; severe symptoms: 27/42, 64%) agreed that the proposed RA could be an alternative to caregivers or HCPs for non-life-threatening situations. Of the participants with mild symptoms or severe symptoms, 0% (0/56) and 9% (4/42), respectively, declined to accept it as an alternative, and the rest (27/98, 28%) were unsure (ie, neutral).

### Preferred Platform

We also asked participants to indicate which platforms (end devices; eg, smartphones, smartwatches, voice assistants [eg, Amazon Alexa, Google Home Mini], and computers [laptops and desktops]) they would prefer for the proposed system. Of the participants, 93% (91/98) chose smartphones, while 17% (16/98) chose smartwatches. Voice assistants and personal computers were ranked in the second and third positions, with 33% (32/98) and 27% (26/98) of the votes, respectively. These preferences inspired us to develop the target RA as a platform-independent (eg, web-based or cloud-based) application so that the target users can have access to the RA on any of their devices.

### Design Feedback

#### Overview

We identified 6 significant aspects as a result of the thematic analysis. Briefly, the mildly infected participants wanted to receive more information about the disease and use an interactive interface, whereas the severely infected participants requested tracking and automation within the system. Both groups thought the system could improve in terms of providing emotional support and specific recommendations for managing the disease. The summary (including a few quotes from participants) of identified aspects are provided in the following sections.

#### (1) Automation

Participants thought that the system could be automated to perform additional functions. Particularly, participants wanted the system to automatically determine participants’ health status and take subsequent actions. They specifically urged for an automatic and robust way of handling medical emergencies without the user’s input:

Since the system has access to bodily measurements, why do users have to initiate the procedure? I guess the system can automatically dispatch a notification to the user alerting about their condition.

P7, Scenario-1

In some cases, patients can’t respond, and the guide should act independently to contact emergency services.

P22, Scenario-2B

#### (2) Information

Participants suggested that the RA should provide users with accurate, up-to-date, and location-specific information. In particular, participants recommended that the RA should be able to provide various kinds of information such as testing appointments and costs:

News Feed (Regularly updated with new Covid-19 Rules including travel restrictions, safety measures, social gathering restrictions etc.).

P1, Scenario-1

Testing strategies vary from country to country; in many places, you can’t just turn up at the test center, but need to phone ahead and make an appointment, or speak to an epidemiologist ...

P5, Scenario-1

#### (3) Tracking

Participants thought that the RA should be able to track users’ actions and data, so future actions can be informed:

It could track progression of COVID-19 symptoms so it could help with future diagnosis.

P37, Scenario-1 and Scenario-2

Tracking of the location for that day if the person went to hospital for the test or not.

P25, Scenario-1

#### (4) Emotional Support

Some participants thought the RA would be useful in providing emotional support to patients. In particular, participants thought that the RA could help them feel less lonely during and after recovery, as it gave the impression that someone was looking after them:

Just wanted to say, I like this feature a lot! The worst part for me was how alone I felt during and after recovery (isolated at home). This kind of thing can be a huge help.

P53, Scenario-2A and Scenario-3

Participants recommended that the ability of the RA to provide emotional support be further improved. Some participants suggested that the RA should allow patients to seek emotional support from their loved ones. Others suggested connectivity with social media groups:

Communication with loved ones may help in reducing PTSD. This feature might be included.

P21, Scenario-3

Other participants thought that the system would not be able to replace a human in terms of providing emotional support:

I think talking to someone on the phone is more helpful than texting into the system.

P34, Scenario-3

#### (5) Personalized Recommendations

Participants thought that the RA’s ability to provide more personalized recommendations to the user could be improved. Participants thought that the system should be able to anticipate the user’s situation and provide help and resources accordingly:

Some medical assistance can be added (eg, medicines, case studies, home remedies) according to a person’s symptoms.

P23, Scenario-2A

Infected person normally loses the ability to taste food and smell, some suggestions can be provided in case the person is frustrated about this.

P11, Scenario-1 and Scenario-2A

Participants also thought that the system should go beyond just recommending to also explaining how these recommendations would be helpful to the patient:

It’s alright to show what to eat, what type of exercise to do, other precautions to take — it can also be explained why these habits will be helping the COVID-19 patient.

P19, Scenario-2A

#### (6) Interactive Interface

Some recommendations revolved around making the interface more fun and interactive to increase users’ engagement with the system:

The exercise tips can be animated instead of still images to make them clear to the person.

P42, Scenario-2A

Participants also suggested that the proposed RA should be updated to include some entertaining features such as fun games and videos, since patients believed that such features can temporarily diverge patients from the stress of the infection:

Can you add some funny video or fun game. I hope this will help a covid 19 positive people to enjoy some little time with joy to think outside of his or her physical condition.

P15, Scenario-2A and Scenario-3

## Discussion

### Principal Findings

This article presents the design validation of a prototypical RA, that targets SARS-CoV-2–infected patients during various stages of the disease. The prototypical RA is the end product of several iterations of finalizing the design requirements (ie, intended users and task identification, interaction dialog design). The RA may also be able to provide COVID-19 patients with immediate assistance. Content and services of the RA may be tailored to address immediate COVID-19 health issues, make recommendations for remedies, and then monitor the patient's condition.

The COVID-19 outbreak led to the development of a substantial number of CAs or VAs, but research reveals that few of them have been validated in terms of their usability or usefulness or the willingness to be used by patients. For example, Ishii et al [13] only showed the design process of a CA-based companion for people in COVID-19 quarantine. In our work, we addressed these gaps by presenting relevant findings about our proposed RA along with the design process. Our proposed RA has the benefit of being relational in creating relationships with the patients from the very beginning of the infection through the recovery stages when compared with state-of-the-art CA development targeting COVID-19. Other CAs of a similar type have primarily been created to focus on just 1 or 2 COVID-19–related problems or to target a particular COVID-19 stage. For instance, Loveys et al [6] proposed a virtual human–based CA that helped individuals by providing remote intervention for stress and loneliness during COVID-19. Another example is from Siedlikowski et al [17], who presented a CA that acted as a self-assessment tool for COVID-19 diagnosis. The key benefit of our proposed RA is that it merged the 4 possible stages of a COVID-19 patient and addressed them as a whole to deliver health care services depending on the patient's present and actual situation.

According to a recent user study [28] on designing CAs to overcome COVID-19 difficulties, study participants asked that CAs play a number of important tasks. Participants in that study expressed a desire for CAs to be able to serve as an information hub for guidance regarding COVID-19 diagnosis, a personal assistant for providing health recommendations based on an understanding of patients' circumstances, and a mental health tool for relieving patients' stress and feelings of loneliness as they go through the self-quarantine and recovery phases. Participants in the design process also desired that the user personas for the CAs combine comfort and trust, the CAs communicate directly with the patients, and the impact of previous interactions with regard to the infection should be considered when adjusting future interactions with the CAs. The design considerations and needs that the study in [28] reported are all adequately handled in our work, and our proposed RA is capable of fulfilling the participants' intended demands.

Despite the fact that this was a small-scale validation study, the results offer important valuable insights on how an RA could be designed more efficiently to aid COVID-19 patients. Overall, the majority of participants thought the proposed RA was helpful during the COVID-19 infection and recovery phases. The strength of this study is that the perceptions of the target users (ie, COVID-19 patients) on the proposed RA's design were evaluated at an early development stage.

We view this research as an anchor project to showcase how an RA can handle different scenarios (ie, testing guidance [Scenario-1], encouraging healthy habits at home [Scenario-2A], handling emergencies at home [Scenario-2B], and postinfection care [Scenario-3]) for a similar pandemic. The evaluation suggests that the proposed RA is a promising intervention to address the explored scenarios. Furthermore, patients were willing to accept it as a reliable alternative to HCPs in non-life-threatening situations.

We used the SUS to determine the usability of the presented prototype. The SUS is graded on a scale of 1 to 100, with a higher score indicating higher perceived usability, and the acceptable average value is 68. When compared with similar kinds of CAs [29-31] available in the literature, our proposed RA earned a lower average SUS score; however, the reasons for their higher SUS scores are a longer interaction period (in some cases, more than 20 days) with CAs and the use of the Wizard-of-Oz approach. In addition, providing directions including suggested tasks and talks during user studies supervised their interactions between the CAs and the participants. Familiarity with the prototypes for a longer time and known interactions influenced and contributed to improved perceptions of those systems, which resulted in higher average SUS scores. However, there is greater familiarity with typical CAs such as chatbots and voice assistants than with RAs having embodiment. This factor also led to a lower perceived usability score regarding our proposed RA.

Despite receiving a usability rating below the acceptable score, the prototype received positive feedback from the participants, and they were willing to accept it as an alternative to HCPs in non-life-threatening situations. Both mildly and severely infected participants expressed interest in using the proposed system, and they thought that it would be easy to use and learn. Furthermore, regardless of infection severity, participants were positive about the RA’s ability to assist COVID-19 patients. It is noteworthy that all severely infected participants thought that the RA was useful in the present form or could be improved to be useful. No severely infected participant thought that the RA was not useful. The recommendations of each group for improving the interface differed from each other, suggesting that the needs of each group were also different. This makes sense because mildly infected participants were recovering at home, and they were less reliant on medical devices such as a ventilator for recovery. This suggests that we need to pay more attention to understanding the specific needs of mildly and severely infected patients to increase the RA’s appeal.

Participatory design by engaging users during the design and implementation of new technology helps the end product satisfy the needs of its intended stakeholders [32-34]. In line with this fact and the impact of empathy in RA design [35], we engaged the participants to share their views on the final product, and we considered their suggestions and agreed to implement several modifications in the final development of the proposed RA. The ultimate goal is to develop a robust RA-based intervention with maximum usability and efficacy at the time of pandemic situations, particularly by gathering feedback for the RA-enabled system as a social companion in guiding and training the users during various health conditions. These findings are consistent with the prior research explorations described in [3,4,14], and our proposed system performed to a greater extent than the chatbot and ECA-based interventions in [8,15,34-36]. Our findings suggest that, in situations when human intervention is not necessary, patients are willing to receive relevant services from RAs that perform the functions of HCPs, provided the RAs are deliberately and appropriately designed and developed.

### Limitations and Conclusion

The study is characterized by an imbalanced ratio between people with mild and severe symptoms. We acknowledge that feedback from more participants with severe symptoms could have provided a more balanced evaluation of the proposed system. Even though participants with severe symptoms participated in the study, their health conditions (eg, weakness, lack of concentration) may have prevented them from responding properly during the study. Finally, because the interactions with the RA were canned and limited, they may not have seemed particularly natural to the participants. Additionally, there is no concrete way to evaluate the interactions between the RA and its users in this study. There are many excellent inventories for assessing patient-doctor or counselor-client interactions [37-39], but there are not enough for evaluating RA-human interaction in health settings. This requirement for a framework to evaluate the dialogs between an RA and its users might be explored by future research.

Only persons who had COVID-19 and recovered were recruited in this evaluation. However, the first scenario (testing guidance) of the RA's tasks was intended for people who were not sure if they had an infection so they could find out how to test themselves. Since our recruitment led to infected people only, it could be implied that the participants started at the first scenario's stage to confirm they were infected. Based on their past experience with COVID-19 testing, the participants responded to the survey questionnaire for the first scenario. However, a limitation of our study was that none of the individuals who participated had never been infected.

Because of the COVID-19 pandemic, it was not possible to conduct the evaluation study in person. For participants to join remotely and globally while keeping their anonymity, we had to make the survey information and link accessible to the general public. However, now that the pandemic is under control, we plan to do an in-person evaluation study with a new group of individuals by following a standard research protocol.

Participants in the evaluation study did participate anonymously at their discretion, and all responses, such as SARS-CoV-2 infection status, were self-reported. Due to these phenomena, it was possible that the respondents' feedback would not be accurate given that we did not obtain any verification information in order to preserve anonymity.

The work discussed in this paper is the beginning of an intervention that has the potential to serve people during a COVID-19–like pandemic. We are currently developing a high-fidelity version of this tool for in situ evaluation with the target population by addressing the limitations of this work.

## Abbreviations

AI: artificial intelligence
CA: conversational agent
CDC: Centers for Disease Control and Prevention
ECA: embodied conversational agent
HCP: health care professional
ICU: intensive care unit
NLU: natural language understanding
PTSD: posttraumatic stress disorder
RA: relational agent
SSML: Speech Synthesis Markup Language
SUS: System Usability Scale
UCD: user-centered design
VA: virtual assistant
